# Prognostic value of platelet to lymphocyte ratio in predicting survival of patients with metastatic castration‐resistant prostate cancer receiving abiraterone acetate: An evidence‐based case report and review of literature

**DOI:** 10.1002/ccr3.2288

**Published:** 2019-07-11

**Authors:** Oliver E. Yausep, Raksheeth Agarwal, Rifqha Aulina, Anthony E. Wijaya, Ilonka Amaia, Addina W. Moekti, Ikhwan Rinaldi, Agus Rizal A. H. Hamid

**Affiliations:** ^1^ Faculty of Medicine University of Indonesia Jakarta Indonesia; ^2^ Department of Internal Medicine, Medical Hematology‐Oncology Division Cipto Mangunkusumo Hospital Jakarta Indonesia; ^3^ Department of Urology Cipto Mangunkusumo Hospital Jakarta Indonesia

**Keywords:** abiraterone, lymphocyte, metastatic Castration‐Resistant Prostate Cancer, platelet, platelet to lymphocyte ratio, prostate cancer

## Abstract

Platelet to lymphocyte ratio (PLR) is a candidate prognostic marker for metastatic castration‐resistant prostate cancer patients receiving abiraterone acetate and evidence demonstrates that a high PLR is associated with poor survival. More studies are required to verify current findings and establish a definite cutoff point.

## 1 INTRODUCTION

Metastatic Castration‐Resistant Prostate Cancer (mCRPC) is an advanced stage of the disease continuum in prostate cancer and has few treatment options including abiraterone. Recently, studies have shown that platelet to lymphocyte ratio (PLR) has prognostic value in predicting the survival of cancer patients. The aim of this review was to evaluate the prognostic value of PLR in predicting the overall survival of mCRPC patients receiving abiraterone. Searches for studies that compared the overall survival of mCRPC patients with high vs low PLR receiving abiraterone were performed across Medline, EMBASE, the Cochrane Library, and Scopus databases. Studies were then matched with our selection criteria to determine inclusion in this report. Search results yielded two retrospective cohort studies reported that a high PLR is associated with a significantly poorer prognosis in mCPRC patients receiving abiraterone. However, both studies had different cutoff values for a high PLR. PLR can be a candidate prognostic marker for mCRPC patients receiving abiraterone. Additional studies are required to verify and reach a consensus for a cutoff for PLR.

Prostate cancer (PCa) is one of the most common causes of disease and death among men, with 1.6 million diagnoses, and 366 000 deaths annually, worldwide.^1^ The World Health Organization ranked prostate cancer as the second leading cause of death due to cancer in developed countries as of 2015. In Indonesia, prostate cancer is the third most common cancer in men. Almost 60% of new PCa cases are already in metastatic stages.^2^ Chemical castration by Androgen Deprivation Therapy (ADT) is the main mode of treatment for metastatic prostate cancer, and this involves inhibiting androgen synthesis.^3^ Unfortunately, even though patients treated with ADT initially demonstrate high response rates, the cancer will inevitably progress to the final stage in the disease continuum known as metastatic castration‐resistant prostate cancer (mCRPC).^4^, ^5^ mCPRPC is defined as tumor growth despite testosterone suppression (<50 ng/dL) and is usually followed by death within 24‐48 months after development of castration‐resistance.^6^, ^7^

Several novel drugs have been shown to prolong survival and improve quality of life in mCPRPC patients, including abiraterone acetate.^6^ Abiraterone acetate works by inhibiting CYP17A1 enzyme involved in the synthesis of testosterone intraprostatic tissue and is currently recommended as one of first‐line treatment for mCPRC.^4^ Despite this, survival of mCRPC patients after abiraterone acetate therapy still varies.^8^ Moreover, abiraterone acetate therapy is associated with high medical costs.^9^ Considering this, it is essential to find predictors of survival in this cohort to better inform patients of their expected survival, allowing them to weigh the costs and benefits of using abiraterone acetate.

Recently, many studies have demonstrated that the progression and prognosis of cancer are also influenced by host systemic inflammatory response. Clinically, this response is evaluated in terms of neutrophil to lymphocyte ratio (NLR), platelet to lymphocyte ratio (PLR) and C‐reactive protein.^5^, ^6^ PLR is calculated by dividing the platelet count by lymphocytes in a complete blood count.^10^ Growing evidence report that high PLR levels predict poor prognosis in various types of cancers.^11^, ^12^, ^13^, ^14^ However, this notion is relatively novel and only a few studies have tested the prognostic value of PLR in advanced stage prostate cancer. The purpose of this case report is to critically analyze the prognostic value of PLR in mCRPC patients receiving abiraterone acetate therapy.

## 2 CASE HISTORY

In 2013, an 80‐year‐old patient was diagnosed with adenocarcinoma of prostate with a PSA level of 0.33 ng/mL and six spots of metastasis on bone scan in Cipto Mangunkusumo Hospital, Jakarta, Indonesia. The patient does not have a history of diabetes, hypertension, or other comorbidities. The patient was started on ADT for 5 years, until present. In February 2018, the PSA level rose to 54.81 ng/mL, and the bone scan now showed eight spots of metastasis. The patient was subsequently diagnosed with metastatic castration‐resistant prostate cancer (mCRPC) and was started on standard dose abiraterone therapy. Given the new advanced diagnosis, the patient asked about his prognosis, specifically how long he has to live. Recent studies show that the inflammatory marker “platelet to lymphocyte ratio” (PLR) has prognostic value in several types of cancers. At diagnosis of mCRPC, the patients’ peripheral blood count shows a platelet count of 385 000/µL and a lymphocyte count of 1200/µL, yielding a PLR of 320.8. However, the prognostic value of this PLR value is still unclear in mCRPC patients.

Does Platelet to Lymphocyte Ratio predict survival as a prognostic indicator in metastatic Castration‐Resistant Prostate Cancer patients treated with Abiraterone Acetate?

## 3 METHODS

### 3.1 Search strategy

A literature search was done in October 2018 using four databases: PubMed, Scopus, EMBASE, and The Cochrane Library. The keywords used are listed in Table 1.

**Table 1:** Search strategy and keywords used

Database	Search terms	Hits
PubMed	(((((((((("Prostate Cancer"[Title/Abstract]) OR "Prostate Cancer"[MeSH Terms]) OR "Prostate Adenocarcinoma"[Title/Abstract]) OR "Prostate Adenocarcinoma"[MeSH Terms]) OR "Prostatic Neoplasms"[Title/Abstract]) OR "Prostatic Neoplasms"[MeSH Terms]) OR CRPC[Title/Abstract]) OR CRPC[MeSH Terms])) AND (((((("Platelet to lymphocyte ratio"[Title/Abstract]) OR "Platelet to lymphocyte ratio"[MeSH Terms]) OR "platelet lymphocyte ratio"[Title/Abstract]) OR "platelet lymphocyte ratio"[MeSH Terms]) OR PLR[Title/Abstract]) OR PLR[MeSH Terms])) AND ((((((survival[Title/Abstract]) OR survival[MeSH Terms]) OR mortality[Title/Abstract]) OR mortality[MeSH Terms]) OR prognosis[Title/Abstract]) OR prognosis[MeSH Terms])	13
Scopus	TITLE‐ABS‐KEY ( "prostate cancer" OR "prostate adenocarcinoma" OR "prostatic neoplasms" OR crpc) AND TITLE‐ABS‐KEY ( "platelet to lymphocyte ratio" OR "platelet lymphocyte ratio" OR plr) AND TITLE‐ABS‐KEY ( mortality OR prognosis OR survival)	21
Cochrane	(“Prostate Cancer” OR “Prostate Adenocarcinoma” OR “Prostatic Neoplasms” OR CRPC):ti,ab,kw AND (“Platelet to Lymphocyte Ratio” OR “Platelet Lymphocyte Ratio” OR PLR):ti,ab,kw AND (Survival OR Mortality OR Prognosis):ti,ab,kw	1
EMBASE	prostate cancer.mp. prostate adenocarcinoma.mp. platelet to lymphocyte ratio.mp. platelet lymphocyte ratio.mp. or platelet lymphocyte ratio/ platelet lymphocyte ratio/ 3 or 4 or 5 survival.mp. mortality.mp. or mortality/ prognosis/ or prognosis.mp. 7 or 8 or 9 prostate neoplasms.mp. 1 or 2 or 11 6 and 10 and 12	22

### 3.2 Eligibility criteria

We included studies performed on mCRPC patients receiving abiraterone acetate that reported dichotomized PLR levels and overall survival. The types of studies included were systematic reviews, meta‐analyzes, and cohort studies. Studies that included mCRPC patients receiving other forms of intervention or those that do not report overall survival as a measure of prognosis were excluded.

### 3.3 Critical appraisal

The selected studies were critically analyzed, by consensus of all authors, using the Critical Appraisal for Prognostic Studies checklist from www.cebm.net which was developed by Oxford University.

### 3.4 Data extraction

The data extracted from each paper included study design, patient characteristics (disease and treatment received), PLR levels, and overall survival.

## 4 RESULT

### 4.1 Search selection

**Figure 1:**
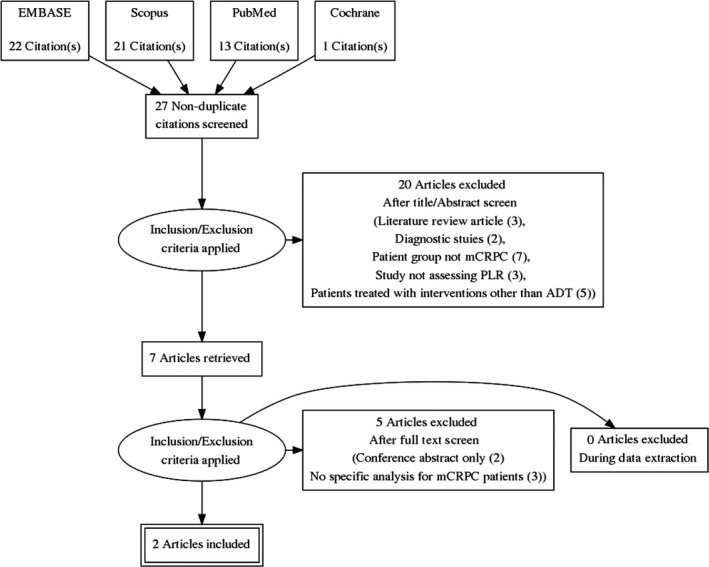
Prisma Diagram. A search across 4 databases yielded 27 non‐duplicate citations. Filtering through these papers using prior determined eligibility criteria yielded 2 viable studies for inclusion

A comprehensive search was done using four different databases with search terms provided in Table 1. Given the specific topic of this report, time restrictions were not applied to increase the sensitivity of the search. Various studies were considered relevant and were further selected based on their fulfillment of the eligibility criteria. The Preferred Reporting Items for Systematic Reviews and Meta‐Analysis (PRISMA) diagram was used to outline the sequential steps in study selection (Figure 1). From the 57 articles collected, 30 duplicates were removed from the initial search result. Subsequently, title and abstract screening were done to eliminate irrelevant articles. With the implementation of the other inclusion and exclusion criteria, and reviewing retrieved full text articles, two articles were included in the final analysis. These were studies done by Martinez et al and Lolli et al.^15^, ^16^

### 4.2 Critical appraisal

To assess the quality of the selected studies, a critical appraisal tool from Oxford CEBM was used. We evaluated the validity, importance, and applicability of the included studies (Table 2). Overall, both studies were appeared to have good quality, although Martinez et al failed to provide confidence intervals for extracted outcome measures.

**Table 2:** Critical appraisal result

	Criteria	Martinez et al^15^	Lolli et al^16^
Validity	Was the defined representative sample of patients assembled at a common (usually early) point in the course of their disease?	Yes	Yes
	Was patient follow up sufficiently long and complete?	Yes	Yes
	Were outcome criteria either objective or applied in a “blind” fashion?	Yes	Yes
	If subgroups with different prognoses are identified, did adjustment for important prognostic factors take place?	N/A	No
Importance	What were the overall results of the study?	Cutoff = 150 Median OS (months) High PLR: 15.9 Low PLR: 27.4 (*P* = 0.005) HR: N/A	Cutoff = 210 Median OS (months) High PLR: 14.4 (CI: 11.2‐17.3) Low PLR: 19.0 (CI: 16.4‐22.3) HR: 1.68 (1.20‐2.35) (*P* = 0.002)
	How precise were the prognostic estimates?	N/A	Precise, as the confidence intervals were narrow
Applicability	Can the results be applied to my patient care?	Yes	Yes
	Were all clinically important outcomes considered?	Yes	Yes
	Are the benefits worth the harm and costs?	Yes	Yes

Note

Both studies were satisfactory in terms of validity, importance, and applicability. Martinez et al did not provide confidence intervals for their results; hence, we were unable to gain insight on their precision.

### 4.3 Study results

Both articles that were selected from the search were retrospective cohort studies. Martinez et al explored the relationship between PLR and the survival rates in 101 mCRPC patients treated with abiraterone, with 45 receiving previous docetaxel therapy. This study reported that high values of PLR (>210) reflected a significantly worse prognosis in terms of overall survival in mCRPC patients (15.9 vs 27.4 months, *P* = 0.005), which was analyzed by logrank test.^15^ Lolli et al conducted a study with 230 mCRPC patients, evaluating the prognostic role of systemic immune‐inflammation index (SII) in mCRPC patients who received abiraterone with previous docetaxel treatment. Lolli et al^16^ also found that a high PLR (>150) is significantly associated with shorter overall survival (14.4 vs 19.0 months, *P* = 0.002), with a hazard ratio (HR) of 1.68 (1.20‐2.35), analyzed by cox proportional hazard regression models (Table 2).

## 5 DISCUSSION

Abiraterone acetate has been shown to prolong survival in mCRPC patients.^6^ However, the response rates to abiraterone are low, thus new biomarkers are required to accurately stratify patients based on their prognostic risk. A high PLR has been shown to predict poor prognoses in various cancers such as hepatocellular carcinoma, breast cancer, urological cancers, and pancreatic cancer.^11^, ^12^, ^13^, ^14^ This is consistent with the results from both included studies which show that patients with a higher PLR had significantly lower overall survival rates, demonstrating the prognostic value of this laboratory marker in mCRPC patients treated with abiraterone.^15^, ^16^ Based on these findings, our patient's PLR, which was 320.8, should be associated with a poorer overall survival.

A high PLR indicates chronic inflammation in cancer patients, which is known to play a major role in carcinogenesis and tumor progression.^17^ The use of peripheral blood count as an indicator of localized or systemic inflammatory event is a well‐established concept.^18^ High platelet levels can be associated with systemic inflammation or spreading of cancer cells via platelet clots.^18^ Platelets have also been implicated in tumor aggressiveness due to their ability to promote neoangiogenesis, increase MMP‐9 secretion, and promote the expression of endothelial adhesion molecules that facilitate tumor cell attachment to metastatic sites.^19^, ^20^ Low lymphocyte levels can indicate impaired activation of adaptive immunity or poor nutritional status.^18^ Unlike platelets, lymphocytes are known to hinder malignant cancer progression, as shown in several studies where high lymphocyte levels increase the survival of patients with multiple cancers.^21^ Therefore, high platelet and/or low lymphocyte levels, as indicated by increased PLR, can contribute to poor prognosis in cancer patients.

Platelet to lymphocyte ratio, which is derived from peripheral blood count, is a relatively cost‐effective and simple test and would be feasible in clinical settings. Furthermore, PCa patients already have their blood routinely collected to monitor PSA levels. However, several factors that could influence PLR are ethnicity, smoking history, chronic infection, hematological disease, and peripheral artery disease.^22^, ^23^ As such, interpretation of this biomarker must be done with caution by taking into account the patients holistic condition.

There are several limitations to this report. Only two studies were able to be included, possibly because the use of PLR as a prognostic marker for prostate cancer is a relatively novel concept. Both included studies are retrospective in nature, which is more sensitive to bias than prospective cohort studies. Additionally, Martinez et al^15^ included patients with and without a history of previous docetaxel therapy, prior to abiraterone, without performing subgroup analyzes between these two groups. Previously, abiraterone was only indicated in mCRPC patients with previous docetaxel therapy; however, recent studies and current guidelines support its use even in chemotherapy naïve patients.^6^, ^24^, ^25^, ^26^ Due to the low number of studies currently available, no consensus on a cutoff value for high PLR or a standardized time period to assess PLR has been reached. Future studies should aim to establish a more specific cutoff point for PLR and a standardized time of assessment. The Prostate Cancer Clinical Trials Working Group recommends that the best time to evaluate progression would be 12 weeks after treatment initiation to ensure adequate drug exposure and anticipate late responses and flare reactions.^27^ More prospective studies need to be done, ideally with an agreed upon cutoff for high PLR and time of measurement, before PLR could officially be used to stratify clinical outcomes. Subgroup analyzes between groups with or without previous docetaxel treatment are also required to identify the effects of prior docetaxel treatment on prognosis. Aside from this, future studies can assess the prognostic power of PLR when combined, with other identified prognostic markers such as bone‐related parameters, alkaline phosphatase, or lactate dehydrogenase.^28^, ^29^ Additional background information on the mechanisms underlying how a high PLR predicts poor prognosis in mCRPC patients also needs to be pursued.

## 6 CONCLUSION

Overall, both included studies report that a high PLR predicts poor overall survival in mCRPC patients receiving abiraterone acetate. PLR can be calculated from routine complete blood count data and hence has potential to be a cost‐effective prognostic marker in this cohort of patients. However, more prospective cohort studies are needed to confirm these results, and a consensus for a cutoff value for PLR must be reached before this marker can be applied in clinical practice.

## CONFLICT OF INTEREST

The authors declare no conflicts of interest in the making of this paper.

## AUTHOR CONTRIBUTIONS

OEY: involved in conception of paper, writing, and critical appraisal. RA: involved in writing and critical appraisal. RA: involved in writing and critical appraisal. AEW: involved in writing and critical appraisal. IA: involved in writing and critical appraisal. AW: involved in writing and critical appraisal. IR: involved in critical appraisal, expert opinion, and current oncology evidence. ARAHH: involved in expert opinion, patient care, and data collection.
